# Outcomes of Metabolic and Bariatric Surgery Among Adolescents and Young Adults in Southern Louisiana

**DOI:** 10.1007/s11695-026-08709-y

**Published:** 2026-07-02

**Authors:** Zubaidah Nor Hanipah, Laura M. Boyer, Katie E. Queen, Amanda E. Staiano, Kimberly L. Drews, Vance L. Albaugh, Peter T. Katzmarzyk, Philip R. Schauer

**Affiliations:** 1 Metamor Institute, Pennington Biomedical Research Center, Baton Rouge, USA; 2 Department of Surgery, Faculty of Medicine and Health Sciences, Universiti Putra Malaysia, Serdang, Malaysia; 3 FMOL Heath Our Lady of the Lake Children′s Heath -Weight and Nutrition Center, Our Lady of the Lake Children’s Hospital, Baton Rouge, USA; 4 Pennington Biomedical Research Center, Baton Rouge, USA; 5 Our Lady of the Lake Regional Medical Center, Baton Rouge, USA; 6 Department of Surgery, Louisiana State University Health Sciences Center New Orleans, New Orleans, USA

**Keywords:** Bariatric surgery, Adolescent, Youth, Complications, Type 2 diabetes, Mortality

## Abstract

**Background:**

Obesity is rising among adolescents and young adults, leading to serious co-morbidities. Bariatric surgery is recommended for carefully selected patients to improve health and quality of life. This study reviews outcomes in adolescents and young adults who underwent bariatric surgery at an accredited center in Louisiana.

**Methods:**

Patients aged 10 to 25 years who underwent bariatric surgery between January 2020 and March 2025 were retrospectively studied.

**Results:**

A total of 76 patients were identified, 80% female, with a mean age of 22.6 ± 2.7 years, weight of 136.8 ± 26.3 kg, and body mass index (BMI) of 49.4 ± 7.6 kg/m^2^. Half of the cohort were African American, and 75% had Medicaid insurance coverage. Comorbidities were type 2 diabetes (32%), biopsy-confirmed metabolic dysfunction-associated steatohepatitis (33%), hypertension (18%), gastroesophageal reflux disease (GERD, 38%), sleep apnea (17%), and dyslipidemia (16%). Procedures included sleeve gastrectomy (59%), Roux-en-Y gastric bypass (37%), and single anastomosis duodenal ileostomy (4%), all performed laparoscopically. Mean operative time was 107.1 ± 46.1 min, and mean hospital stay was 1.5 ± 1.2 days. Thirty-day complications occurred in 5%, with 5% readmissions and 4% requiring reoperation. There was no mortality at 30 days or 1 year. Mean follow-up was 16 months (range 1–48). Mean percentage total weight loss (TWL%): 29.4% ± 9.9% at 1 year (69% follow‑up), 29.7% ± 13.1% at 2 years (57%, follow-up) and 32.0% ± 13.9% at 3–5 years (50%, follow-up). Among patients with follow-up data, diabetes remission occurred in 94% (15/16), while improvements were observed in hypertension (67%, 8/12), dyslipidemia (67%, 4/6), and GERD (64%, 7/11).

**Conclusion:**

Among adolescents and young adults from Southern Louisiana with available follow‑up data, bariatric surgery was associated with favorable short‑ to mid‑term safety outcomes and improvements in weight and obesity‑related comorbidities.

**Supplementary Information:**

The online version contains supplementary material available at 10.1007/s11695-026-08709-y.

## Key Points

- Bariatric surgery among adolescents and young adults in Southern Louisiana demonstrated low perioperative risk (5% complication rate), with no mortality at 30 days or 1 year
- Patients achieved significant and durable weight loss (29–32% at 1–5 years) and high rates of improvement or remission in obesity-related comorbidities, including type 2 diabetes (94%), hypertension and dyslipidemia (67%), and GERD (64%).
- The cohort was largely Medicaid-insured (75%), highlighting the importance of accessible bariatric care and supporting surgery as a treatment option regardless of age or socioeconomic status.

**Supplementary Information:**

The online version contains supplementary material available at 10.1007/s11695-026-08709-y.

## Introduction

The escalating prevalence of obesity among adolescents and young adults in the United States continues to pose a major public health challenge. National Health and Nutrition Examination Survey (NHANES) data indicate that obesity prevalence among adolescents aged 12–19, has reached 23%, with severe obesity increased from 6.1% (2017–2020) to 8.9% in 2023 [1]. This surge in adolescent obesity is associated with a wide range of medical, psychological, and economic consequences. Chronic conditions such as type 2 diabetes (T2D), obstructive sleep apnea (OSA), metabolic dysfunction-associated fatty liver disease (MAFLD), and cardiovascular disease often persist into adulthood [2, 3].

Metabolic and bariatric surgery (MBS) has emerged as a safe and effective intervention for adolescents with severe obesity. Supported by guidelines from the American Academy of Pediatrics (AAP) and the American Society for Metabolic and Bariatric Surgery (ASMBS), MBS offers morbidity and mortality outcomes comparable to those seen in adults [2–4]. Despite these endorsements, MBS remains underutilized among adolescents. Between 2010 and 2017, approximately 1,300 to 1,900 adolescents underwent MBS annually in the U.S. [5]. From 2015 to 2018, the utilization rate was 1.81 per 1,000 youth compared to 5.56 per 1,000 adults (*P* < 0.001) [6]. However, utilization has increased between 2018 and 2021, from 800 (2018) to almost 1400 cases in 2021 [7].

Though adolescence is generally defined as ages 10–19 years, a definition supported by the World Health Organization, some experts and organizations advocate for an expanded definition that goes up to age 24 years, arguing that neurological development of the pre-frontal cortex is not complete until around this age [8, 9]. In alignment with this broader developmental framework, this study evaluates the safety and effectiveness of MBS across extended adolescence and young adulthood (ages 10–25 years) by analyzing patient demographics, comorbidities, perioperative outcomes, and postoperative weight and comorbidity resolution from an ASMBS accredited metabolic surgery center.

## Methods

### Study Population

This retrospective study was conducted at Metamor Institute, Penington Biomedical Research Center and Our Lady of the Lake Regional Medical Center (OLOLRMC) in Baton Rouge, LA, after obtaining institutional review board approval (2024–058-PBRC). Patients aged 10–25 years underwent bariatric procedures in a single center between January 2020 and March 2025. Patients were generally referred by their pediatrician, primary care physician, or self-referred. Patients with a body mass index (BMI) over 35 kg/m^2^, without active substance use or unstable psychiatric conditions, were carefully evaluated by a multidisciplinary team of surgeons, psychologists, obesity medicine specialists/pediatricians, and dieticians to determine eligibility for surgery.

### Surgical Procedures, Preoperative Patient Preparation and Postoperative Management

Procedure selection was individualized based on a risk–benefit assessment, considering BMI, comorbidities such as T2D, gastroesophageal reflux disease (GERD), perioperative risk factors, and the patient’s ability to adhere to follow-up care [2, 10]. Preoperative evaluation for MBS consisted of coordinated multidisciplinary assessments, including evaluations and clearance by the bariatric surgeon, obesity medicine/pediatric specialists, dietitian and behavioral health specialist/clinical psychologist, physical examinations, laboratory studies and diagnostic testing, and confirmation of patient and family readiness to ensure safety, understanding of risks and benefits, and adherence to postoperative care [10]. Additionally, all patients completed standardized preoperative preparation which included structured educational materials on diet, physical activity, and behavioral strategies delivered during clinic visits and reinforced throughout follow‑up.

Prophylactic antibiotics (first-generation cephalosporins or alternatives for penicillin-allergic patients) were administered, and venous thromboembolism (VTE) prevention included pneumatic compression devices and postoperative chemoprophylaxis. Prophylactic cholecystectomy was not routinely performed. All bariatric procedures, including Roux-en-Y gastric bypass (RYGB), sleeve gastrectomy (SG) and single anastomosis duodenal-ileal anastomosis with sleeve gastrectomy (SADI-S) were performed laparoscopically. Post operative and post-discharge VTE prophylaxis are given as needed [2, 10].

In the postoperative period, patients are advised to follow a low calorie, high protein diet along with bariatric multivitamin supplementation. Activity restrictions are generally limited to avoiding heavy lifting for the first month, after which gradual progression to regular physical activity and strengthening exercises is encouraged. Patients are scheduled for clinic follow up visits every three months during the first postoperative year, every six months in the second year, and annually thereafter. Laboratory evaluations and medication adjustments are performed as needed during these visits.

### Data Collection

Preoperative data included demographic characteristics, the presence of obesity-related comorbidites, including hypertension, dyslipidemia, T2D, OSA, cardiovascular disease, GERD, history of thromboembolism, and metabolic dysfunction–associated steatohepatitis (MASH). Information was also collected on medications used for the management of these conditions. The preoperative data was extracted from the Metabolic and Bariatric Surgery Quality Improvement Project (MBSAQIP) database. Preoperative weight is the closest weight to surgery (within 30 days before surgery).

Perioperative parameters, length of hospital stay, and morbidity and mortality rates at 30-days and 1-year post-procedure were extracted. Patients’ weight, BMI, percentage of total weight loss (%TWL), blood pressure, glycated hemoglobin (HbA1c) and fasting lipid profile were recorded preoperatively and at postoperative follow-up intervals of 1 year, 2 years, and 3–5 years.

A patient flow diagram based on weight loss outcomes is available as a supplementary Fig. 1.

### Statistical Analysis and Calculations

Data were summarized as the mean and standard deviation (SD) for continuous variables and as counts and frequency for categorical variables. Patient follow-up visits were based on their annual outpatient clinic visits and any encounters recorded in their electronic medical record (EMR). Clinic staff reached out to the patients via internal EMR messaging, phone and mail to engage for follow up.

Percentage total weight loss (%TWL) was calculated as [(operative weight – follow-up weight)/operative weight)] × 100. Remission of diabetes was defined as follows: HbA1c level < 6.5% or Fasting blood glucose (FBG) < 7.0 mmol/L (126 mg/dL) without the use of an oral hypoglycemic agent, Glucagon‑Like Peptide‑1 Receptor Agonist (GLP‑1RA) or insulin therapy. Glycemic improvement was defined as a reduction in HbA1c by > 1% or FBG by > 1.4 mmol/L (25 mg/dL), or any reduction in HbA1c and FBG accompanied by a decrease in antidiabetic medication requirements [10–12].

Hypertension control was defined as blood pressure (BP) < 140/90 mmHg with or without medication. Dyslipidemia control included low-density lipoprotein (LDL) < 2.6 mmol/L (100 mg/dL), high-density lipoprotein (HDL) > 1.0 mmol/L (40 mg/dL) in men or > 1.2 mmol/L (50 mg/dL) in women, and triglycerides < 1.7 mmol/L (150 mg/dL) with or without medications. We used the definition of control according to the American Diabetes Association criteria with or without medication use [12, 13].

## Results

A total of 76 patients aged 10–25 were identified (Table 1). Sixty-one (80.3%) were female, with a mean age of 22.6 (± 2.7; range 12–25) years. Among the 76 participants, 39 (51.3%) were African American, 36 (47.4%) were White, and 1 (1.3%) identified as another race. The majority had insurance coverage by Medicaid (*n* = 57, 75.0%) while the remainder had commercial insurance (*n* = 14, 18.4%), Medicare (*n* = 2, 2.6%), Tricare (*n* = 1, 1.3%), or cash pay (*n* = 2, 2.6%). The preoperative mean body weight of 136.8 ± 26.3 kg, and mean BMI of 49.4 ± 7.6 kg/m^2^. Comorbidities at baseline initial visit included GERD based on clinical symptoms and therapy (*n* = 29, 38.2%), biopsy proven MASH (*n* = 25, 32.9%), T2D (*n* = 24, 31.6%), hypertension (*n* = 14, 18.4%), sleep apnea (*n* = 13, 17.1%), and dyslipidemia (*n* = 12, 15.8%).

**Table 1:** Patients’ demographics and baseline parameters (*N* = 76)

Characteristics	Value
Age, mean (± SD)	22.6 (± 2.7)
Age category: n (%)	
12 to ≤ 18 years	9 (11.8%)
> 19 to 25 years	67 (88.2%)
Sex, n (%)	
Female	61 (80.3%)
Male	15 (19.7%)
Functional status, n (%)	
Independent	75 (98.7%)
Need assistance	1 (1.3%)
Nonsmoker, n (%)	74 (97.4%)
Co-morbidities, n (%)	
GERD	29 (38.2%)
Biopsy proven MASH	25 (32.9%)
Type 2 Diabetes:	24 (31.6%)
Without insulin	23 (30.3%)
With insulin	1(1.3%)
Hypertension	14 (18.4%)
Dyslipidemia	12 (15.8%)
Sleep Apnea	13 (17.1%)
ASA 2 *	15 (19.7%)
ASA 3 **	61 (80.2%)
Body Weight (kg), mean (± SD)	136.8 (± 26.3)
BMI kg/m^2^, mean (± SD)	49.4 (± 7.6)
Systolic BP mmHg, mean (± SD)	118.6 (13.4)
Diastolic BP mmHg, mean (± SD)	80.1 (± 10.5)

Abbreviations: *SD* standard deviation; *GERD* gastroesophageal reflux disease; *MASH* metabolic dysfunction-associated steatohepatitis; *BMI* body mass index; *ASA* American Society of Anesthesiologists: Physical Status Classification System definitions for *ASA II-Patient with Mild Systemic Disease **ASA III-Patient with Severe Systemic Disease

All bariatric procedures were primary and performed laparoscopically: SG (59%), RYGB (37%), and SADI-S (4%) (Table 2). The majority of patients with T2D underwent SG (*n* = 15), followed by RYGB (*n* = 8), with one patient who had SADI-S. The mean operative time was 107.1 ± 46.1 min, and the mean length of stay was 1.5 ± 1.2 days. Four patients (5%) experienced 30‑day postoperative complications, including one Clavien–Dindo Grade II event (nausea and vomiting requiring intravenous fluids) and three Grade IIIb events requiring surgical intervention under general anesthesia: staple line bleeding requiring transfusion and clot evacuation, cholecystitis requiring cholecystectomy, and a SADI‑S duodenal stump leak with bilious peritonitis necessitating peritoneal washout and primary repair (Table 2). Three patients (4%) required reoperation, two of which occurred in the immediate postoperative period. Four patients (5%) had unplanned readmissions. There was no mortality at 30 days or 1 year.

**Table 2:** Operative outcomes (*n* = 76 patients)

Characteristics	Value
Bariatric and Metabolic Surgery: n (%)	
Sleeve gastrectomy	45 (59%)
Roux-en-Y gastric bypass	28 (37%)
SADI-S	3 (4%)
Laparoscopic, n (%)	58 (76%)
Robotic assisted, n (%)	18 (24%)
Operative time, minutes, mean (± SD)	107.1 (± 46.1)
Length of Hospital stay, day, mean (± SD)	1.5 (± 1.2)
30-day complications: n (%)	4 (5%)
• Stapler line bleeding requires 2 pints blood transfusion and clot evacuation	1
• Cholecystitis (cholesterosis of the gallbladder wall) requiring cholecystectomy	1
• SADI- Duodenal stump leak (0.5 cm) with bilious peritonitis—peritoneal washout and primary repair of the stump leak	1
• Nausea/vomiting requiring intravenous fluids	1
30-day Readmission: n (%)	4 (5%)
• Cholecystitis (cholesterosis of the gallbladder wall) requiring cholecystectomy	1
• Non-specific abdominal pain	1
• Nausea/vomiting requiring IV fluids	1
• MVA with mandibular fracture	1
30-day Reoperation: n (%)	3 (4%)
30-day mortality	0
1-year mortality	0

Abbreviations: *SADI-S* single anastomosis duodenal-ileal anastomosis with sleeve gastrectomy; *MVA* motor vehicle accident

The mean follow-up period was 16 (range 1–48) months (Table 3). The mean percentage of total weight loss was 29.4% ± 9.9% at 1 year (69% follow‑up; 95% Confidence interval (CI) 26.4–32.5), 29.7% ± 13.1% at 2 years (57% follow‑up; 95% CI 24.3–35.1), and 32.0% ± 13.9% at 3–5 years (50% follow‑up; 95% CI 24.0–40.0). Overall, 93.5% (43/46) of patients achieved ≥ 10% TWL, 91.3% (42/46) achieved ≥ 15% TWL, and 80.0% (37/46) achieved ≥ 20% TWL at the last available follow‑up.

**Table 3:** Weight changes based on bariatric procedures

				1 year		2 years		3–5 years	
Bariatric procedures	Total, N (%)	Baseline BMI (kg/m^2^)	Mean Baseline BW (kg)	n (Present at FU)/ N (eligible for FU), (%)	Mean TWL% (SD)	n (Present at FU)/ N (eligible for FU), (%)	Mean TWL% (SD)	n (Present at FU)/ N (eligible for FU), (%)	Mean TWL% (SD)
All	76 (100%)	49.4	136.8	41/59 * (69%)	29.4 (± 9.9)	24/42 (57%)	29.7 (± 13.1)	14/28 (50%)	32.0 (± 13.9)
SG	45 (59%)	50.7	139	21/37 (57%)	27.6 (± 5.9)	11/25 (44%)	27.6 (± 14.1)	6/17 (35%)	33.8 (± 17.0)
RYGB	28 (37%)	47.2	132	19/21 (90%)	31.7 (± 12.9)	13/16 (81%)	31.5 (± 12.5)	8/10 (80%)	30.6 (± 12.1)
SADI-S	3 (4%)	51	148	1/1 (100%)	25.2	0/1 (0%)	-	0/1 (0%)	-

Abbreviations: *FU* follow-up; *SD* standard deviation; *BW* body weight; *TWL%* total weight loss percentage; *SG* sleeve gastrectomy; *RYGB* Roux-en-Y gastric bypass; *SADI* S single anastomosis duodenal-ileal bypass with sleeve gastrectomy

^*^ 17 patients with < 1 year follow up

Of the 24 patients with T2D, 6 were using GLP‑1RA therapy at baseline. At last follow‑up after surgery, only one patient remained on a GLP‑1RA. Among the 16 patients with available follow‑up data, 15 (93.8%) achieved diabetes remission. The mean HbA1c for the 16 patients with available follow‑up data was 6.2 ± 1.3% at baseline and 5.3 ± 0.3% at follow‑up. The remaining patient demonstrated significant clinical improvement. This patient had been on both insulin and GLP‑1RA‑1RA therapy before surgery and was able to discontinue insulin postoperatively, while continuing GLP‑1RA treatment at last follow‑up.

GERD remission/improvement was observed in 7 out of 11 patients with available data at their final follow-up. Improvement of hypertension and dyslipidemia occurred in 8 out of 12 and 4 out of 6 patients with available follow-up data, respectively. However, for patients had biopsy-proven MASH at the time of bariatric surgery (*n* = 25), post-operative resolution or improvement could not be assessed, as routine repeat liver biopsy was not performed following surgery.

## Discussion

The demand for MBS is increasing, especially among adolescents with severe obesity, who comprise about 9% of the U.S. population [1, 7, 14]. This trend towards advanced treatment options such as MBS reflects the advances in minimally invasive techniques, multidisciplinary care, and improved long-term outcomes [2, 14]. Our experience confirms that MBS is safe and effective during the ages of extended adolescence (ages 12–25 years). Across all MBS procedures, patients with available follow‑up achieved approximately 29–32% total weight loss over 1–5 years, and SG accounted for 59% of procedures performed. T2D remission was achieved in 94% of patients with baseline diabetes who had data available at their last follow-up. Additionally, both hypertension and dyslipidemia improved in 67% of patients with available data at the final follow‑up.

Perioperative safety is a primary concern in adolescent MBS. In our cohort, the average hospital stay was 1.5 days. We observed four (5%) complications: two occurred in the immediate postoperative period and required surgical intervention, while the remaining two arose within 30 days post-surgery. The 30-day reoperation rate was 4%. In the Teen-Longitudinal Assessment of Bariatric Surgery (Teen-LABS) study [15], which evaluated 242 adolescents undergoing bariatric procedures, major complications necessitating reoperation or posing life-threatening risks were reported in 9% of RYGB patients, 5% of SG patients, and 7% of those who underwent adjustable gastric banding (AGB). Minor complications were observed in 17% (RYGB), 12% (SG), and 7% (AGB) patients, with surgical site infections and urinary tract infections being the most common In a meta-analysis of 16 studies evaluating postoperative complications and mortality after metabolic and bariatric surgery, Wu et al. [16] reported a 4.6% readmission rate and a 4.5% reoperation rate, most commonly following adjustable gastric banding. Iron and vitamin B12 deficiencies were among the most commonly reported long-term complications. The analysis identified ten deaths (0.2%) overall, including one suicide 45 months after surgery in a patient with bipolar disorder, one myocardial infarction at 54 months, one case of infectious colitis after gastric bypass, one death from hypoglycemia at three years, two drug overdoses at four years, and four deaths unrelated to bariatric surgery. In our series, the readmission rate was 4% (3 of 76 patients), with no mortality reported through the last follow-up.

In our study, the mean preoperative BMI was 49 kg/m^2^. Among patients with available follow‑up data, the mean percentage total weight loss was 29% at 1 year, 30% at 2 years, and 32% at 3–5 years. Cruz et al. [17] reported a 31% total weight loss at a mean follow‑up of 14 years in 96 adolescents who underwent MBS (91% RYGB, 9% AGB). Similarly, Goldenshluger et al. [18] observed a 32% total weight loss at a mean follow‑up of 11 years among 31 adolescents treated with SG. Major prospective studies such as Teen-LABS, Follow-up of Adolescent Bariatric Surgery at 5 Plus Years (FABS 5 +) and Swedish Adolescent Morbid Obesity Surgery (AMOS) also reported sustained weight loss with mean BMI reductions of 13–17 kg/m^2^ and 26–29% reductions in body weight at five years or more [15, 19–24].

Metabolic and bariatric surgery has demonstrated significant improvements in co-morbidities such as T2D, hypertension, and dyslipidemia in both adolescents and adults. A meta-analysis of 24 cohort studies involving 4,970 adolescents (aged 12–21 years, BMI 39–59 kg/m^2^) reported remission rates of 90% for T2D, 81% for hypertension, and 77% for dyslipidemia [16]. In our cohort, one-third of patients had T2D at baseline. Among the 16 patients with available follow-up data, 94% achieved resolution after MBS, and the remaining patient discontinued insulin therapy and demonstrated clinical improvement. Hypertension and dyslipidemia were present preoperatively in 18% and 16% of patients, respectively. On average, patients with hypertension were taking two antihypertensive drugs prior to surgery. At final follow-up, 67% of those with available data showed remission of hypertension and improvement in dyslipidemia.

In contrast to MBS, lifestyle interventions in the form of diet and exercise as well as pharmacotherapy in adolescents with severe obesity are less invasive but less effective. Over 52 weeks, intensive dietary interventions combined with behavioral therapy produced a − 0.28 reduction in BMI z-score and a 9–10% decrease in BMI relative to the 95th percentile [25]. Until recently the only FDA-approved obesity medications for adolescents aged 12 years and above were orlistat, phentermine/topiramate) and liraglutide. These medications were associated with modest short-term weight loss of approximately 5–7%, which is inadequate for managing severe obesity [26, 27].

In December 2022, semaglutide, the first second generation GLP‑1RA, was approved by the FDA for adolescents 12 years and older with obesity, which produces an average total weight loss of 10% at 68 weeks [28]. Semaglutide and perhaps other second generation GLP‑1RA (Tirzepatide and Orforglipron, currently being studied age 12 years and up) represent a tremendous advance in obesity pharmacotherapy in adolescents. The long-term weight loss in adolescents for these promising agents has yet to be evaluated in adolescents with more severe obesity (> BMI 40 kg/m^2^). MBS, on the other hand, has decades of data that show that it achieves 25–35% total weight loss sustained for 10 years, along with significant rates of remission of T2D, hypertension, and sleep apnea, and a reduction in long-term mortality risk [29, 30].

Although effective, both pharmacotherapy and surgery carry specific risks. MBS inherently involves operative complications such bleeding, infection, nutrient deficiencies and require lifelong follow-up, while medications require ongoing use and may cause gastrointestinal side effects or rare events such as pancreatitis [27]. Access remains a major barrier for both options. GLP‑1RA medications often cost from $150 to over $1,000 per month and are rarely covered by insurance for weight loss, limiting availability for many families [3, 5]. Although MBS has higher initial costs compared to GLP‑1RA, it results in significantly greater weight loss and lower ongoing expenses over two years in patients with class II–III obesity [31]. Nevertheless, it remains underutilized even among eligible patients [2]. Significant disparities persist, with minority and socioeconomically disadvantaged youth experiencing markedly lower access rates [32, 33].

According to AAP 2023 pediatric obesity guidelines, pharmacotherapy is recommended in conjunction with intensive health behavior lifestyle treatment for adolescents aged ≥ 12 years with obesity [4]. Bariatric surgery in conjunction with health behavior lifestyle treatment is indicated for adolescents ≥ 12 years with class 3 obesity (BMI ≥ 40 kg/m^2^ or ≥ 140% of the 95th percentile) or class 2 obesity (BMI ≥ 35 kg/m^2^ or ≥ 120% of the 95th percentile) with serious comorbidities such as T2D or obstructive sleep apnea [2–4]. Surgery is most effective for severe (class II–III) obesity with progressive co-morbidities or when other therapies fail, while obesity medications may be more appropriate for less severe (Class I or II) obesity as adjuncts to lifestyle therapy and/or pre- and post-operative MBS management. Regardless of modality, comprehensive obesity treatment should be individualized within a multidisciplinary model, considering BMI, comorbidities, psychosocial readiness, and family preferences, and should always include health behavior and lifestyle counseling as a foundation [4, 34].

This study contributes to the growing evidence that MBS is safe and effective in adolescents and young adults with severe obesity, demonstrating sustained weight loss across procedures with up to 5- years of follow-up data previously published. Although we only had 50% cohort retention over a five-year period, this retrospective review offers a real-world data in this population. However, several limitations should be acknowledged. The single‑center retrospective design, modest sample size, and relatively short mean follow‑up of 16 months limit generalizability. Most notably, follow‑up attrition represents a major source of potential bias. Approximately half of the original cohort did not return for longitudinal follow‑up, and the reasons for loss to follow‑up are unknown. As a result, the observed weight‑loss outcomes likely reflect a selected subgroup of patients. This substantial loss to follow‑up also limits the ability to compare procedure‑specific outcomes, evaluate late complications, and assess long‑term comorbidity resolution. Missing clinical and laboratory data were primarily due to loss to follow-up and patients receiving care outside our institution. Consequently, these findings should be interpreted as exploratory and hypothesis‑generating rather than definitive. Despite outreach efforts through phone calls (3 calls per missed visit) and mailed reminders, follow‑up beyond three years remained suboptimal at approximately 60%, which is notably lower than the follow‑up rates of up to 85% reported in other prospective studies in this population [21, 29].

To address these challenges, our center has recently implemented several enhanced retention strategies aimed at improving long‑term engagement. These include automated EHR‑based reminders delivered through MyChart messaging, supplemental reminder phone calls, mailed letters, and coordinated follow‑up with pediatric obesity medicine providers to support continuity of care as patients transition to adult services. These strategies are anticipated to facilitate more consistent communication, reduce missed visits, and improve patient adherence to postoperative monitoring. Incorporating additional technology‑driven approaches such as mobile applications that allow patients to upload weight, laboratory results, and symptom updates, alongside telemedicine consultations may further enhance accessibility and long‑term retention. Ultimately, larger multicenter prospective studies with standardized, technology‑supported follow‑up protocols will be essential to more comprehensively evaluate long‑term outcomes of MBS in adolescents and young adults.

## Conclusion

In this single‑center cohort, outcomes among patients with available follow‑up suggest that MBS can be delivered safely and effectively in socioeconomically diverse young individuals. However, because only 12% of the cohort was younger than 18 years, these findings should not be viewed specific to only young adolescents; rather, they reflect results across the broader adolescent–young adult age group (≤ 25 years). Additionally, incomplete follow‑up limits our ability to comprehensively evaluate long‑term comorbidity resolution and compare weight‑loss outcomes across procedures. Therefore, the observed improvements in comorbidities and procedure‑related weight loss should be interpreted as indicative rather than conclusive.

Optimal outcomes in this younger population were likely supported by appropriate patient and procedure selection, alongside coordinated multidisciplinary care. Larger prospective studies with more complete follow‑up are needed to strengthen conclusions and to guide decision‑making regarding patient eligibility and procedural selection in this patient population.

## Supplementary Information

Below is the link to the electronic supplementary material.

Supplementary file1 (DOCX 132 KB)

## Data Availability

No datasets were generated or analysed during the current study.
