# Supplementary material for: Outcomes of Metabolic and Bariatric Surgery Among Adolescents and Young Adults in Southern Louisiana
Source: Obes Surg. 2026 Jul 2;36(8):4096–103. doi: 10.1007/s11695-026-08709-y (PMC13429526; doi:10.1007/s11695-026-08709-y)

**Supplement Table 1. Weight Loss and Comorbidity Outcomes with Denominator Tracking**

**Table1A. Weight Loss Outcomes by Follow‑up Time**

**Table1A. Weight Loss Outcomes by Follow‑up Time**

| **Follow‑up time** | **Eligible for follow‑up, N** | **With available follow up data, *n* (%)** | **Mean %Total Weight Loss (SD)** | **95% Confidence interval** |
| --- | --- | --- | --- | --- |
| 1 year | 59 ** | 41 (69.5%) | 29.4 ± 9.9 | 26.4-32.5 |
| 2 years | 42 | 24 (57.1%) | 29.7 ± 13.1 | 24.3–35.1 |
| 3–5 years | 28 | 14 (50.0%) | 32.0 ± 13.9 | 24.0–40.0 |

** Seventeen patients were excluded from 1‑year analysis due to <12 months of follow‑up.

**Table 1B. Comorbidity Outcomes at Last Available Follow‑up**

| **Comorbidity** | **Baseline with condition, N** | **With follow‑up data, *n*** | **Patients with outcome, *n* (%)** |
| --- | --- | --- | --- |
| Type 2 diabetes | 24 | 16 | Remission: 15 (94%)  Improvement: 1 (6%)  Data not available: 8 |
| Hypertension | 14 | 12 | Remission: 0  Improvement: 8 (67%)  No improvement: 4  Data not available: 2 |
| Dyslipidemia | 12 | 6 | Remission:0  Improvement: 6 (67%)  Data not available: 6 |
| GERD | 29 | 11 | Improvement: 11  Data not available: 18 |
| MASH |  | Not available (no repeat liver biopsy was performed) |  |

**Supplement Figure 1: CONSORT-style patient flow diagram based on weight loss outcomes**


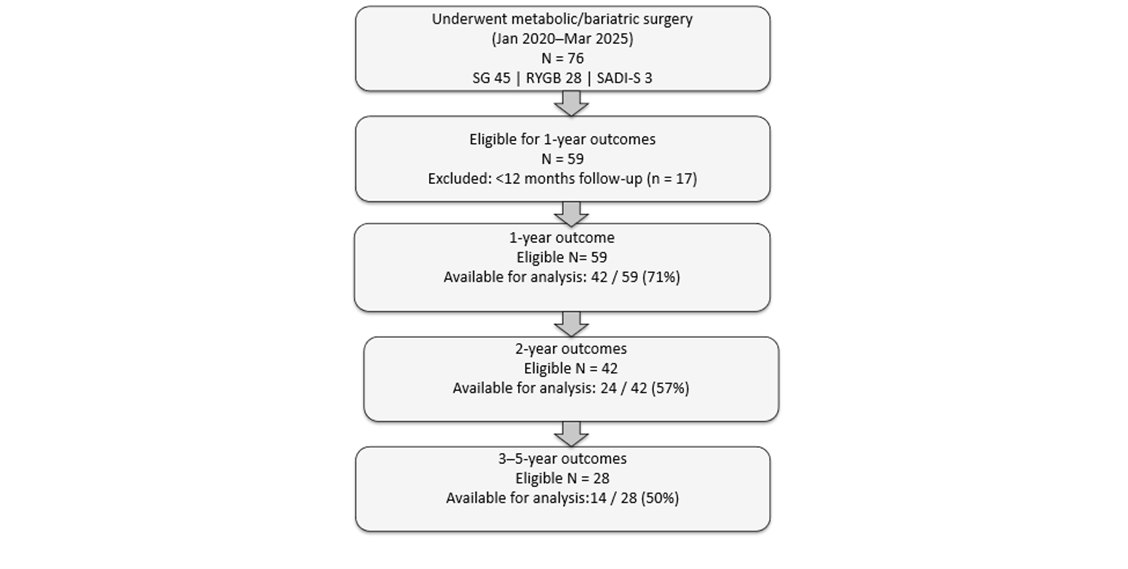

Supplement: Supplementary file 1 — Supplementary file1 (DOCX 132 KB) [file 11695_2026_8709_MOESM1_ESM.docx]
